# Biochar as a low-cost, eco-friendly, and electrically conductive material for terahertz applications

**DOI:** 10.1038/s41598-021-98009-5

**Published:** 2021-09-16

**Authors:** Woongkyu Park, Hyuntae Kim, Hajung Park, Soobong Choi, Sung Ju Hong, Young-Mi Bahk

**Affiliations:** 1 Intelligent Sensor Convergence Research Center (ISCRC), Incheon National University, 22012 Incheon, Republic of Korea; 2 Department of Physics, Incheon National University, 22012 Incheon, Republic of Korea; 3 Division of Science Education, Kangwon National University, 24341 Chuncheon, Republic of Korea

**Keywords:** Terahertz optics, Sustainability

## Abstract

We investigate conducting characteristics of biochar derived from the pyrolysis of a paper at terahertz frequencies. Paper is annealed under temperatures ranging from 600 to 1000 °C to modify structural and electrical properties. We experimentally observe that the terahertz conductivity increases above 10^2^ S/m as the annealing temperature increases up to 800 °C. From structural characterization using energy-dispersive X-ray spectroscopy, Fourier-transform infrared spectroscopy, Raman spectroscopy, X-ray diffraction, and X-ray photoelectron spectroscopy, we confirm that more graphitic biochars are produced in high annealing temperature, in agreement with the improvement of terahertz conductivity. Our results show that biochar can be a highly promising candidate to be used in paper-based devices operating at terahertz frequencies.

## Introduction

Paper is a cost-effective, abundant, disposable, and flexible material, therefore it is widely used in qualitative and simple diagnoses such as litmus tests^1^ and pregnancy tests^2^. Despite the advantages, poor electrical conductivity prevents it from being a sensing platform. Biochar obtained by annealing without oxygen can be a breakthrough for inducing electrical properties. Furthermore, its chemical stability under ambient conditions leads to various applications such as supercapacitor^3^, sensor^4^, water purification^5^, and conducting ink^6^. Therefore, the electrical evaluation of the biochar is of significance in the aspect of both fundamental and applicable points of view.

Terahertz time-domain spectroscopy is a representative tool for characterizing the electrical properties of material ranging from metal to dielectric in a non-contact manner. Especially, the terahertz wave can easily penetrate dielectric materials which are opaque in the visible and near-infrared regime. Therefore, various studies related to paper-based devices have also been conducted in terahertz optics; e.g., terahertz zone plate^7^, terahertz photonic crystal^8^, and terahertz metamaterial^9,10^. However, the previous works require an additional metal patterning process to realize terahertz devices on the paper-based substrates. Such a process generally needs expensive ingredients and generates pollutants, which contradicts using paper-based substrates as an inexpensive and eco-friendly sensing platform. In that regard, investigating the biochar in the terahertz regime is attractive to search low cost and eco-friendly materials.

Even though the biochars can be a potential candidate as an alternative, conductive material at the terahertz frequencies, there are few studies on their optical properties. Lepodise et al. investigated the absorption characteristics of aromatic carbon structures in biochars in the regime of 10 THz^11^. In addition, Pogson et al. investigated the capability of the terahertz spectroscopy to detect or screen the various biochar components^12^. However, the conducting characteristics of biochar at the low terahertz frequency range (below 2 THz) are not studied yet. Since extensive studies for device application in the frequency regime are being actively conducted, investigating the terahertz electrical conductivity of biochar in that range is of significance.

Here, we investigate the conducting characteristics of the biochar at low terahertz frequencies (0.7–1.5 THz). The biochar is prepared by annealing under a nitrogen (N_2_) atmosphere at various temperatures. We confirm that the annealing process improves electrical conductivity in terms of terahertz time-domain spectroscopy. By harnessing structural analysis, we reveal that the origin of electrical conductivity enhancement of the biochar. We attribute the enhancement to the crystallization of carbon elements in the fibril network.

### Results and discussion

**Figure 1:**
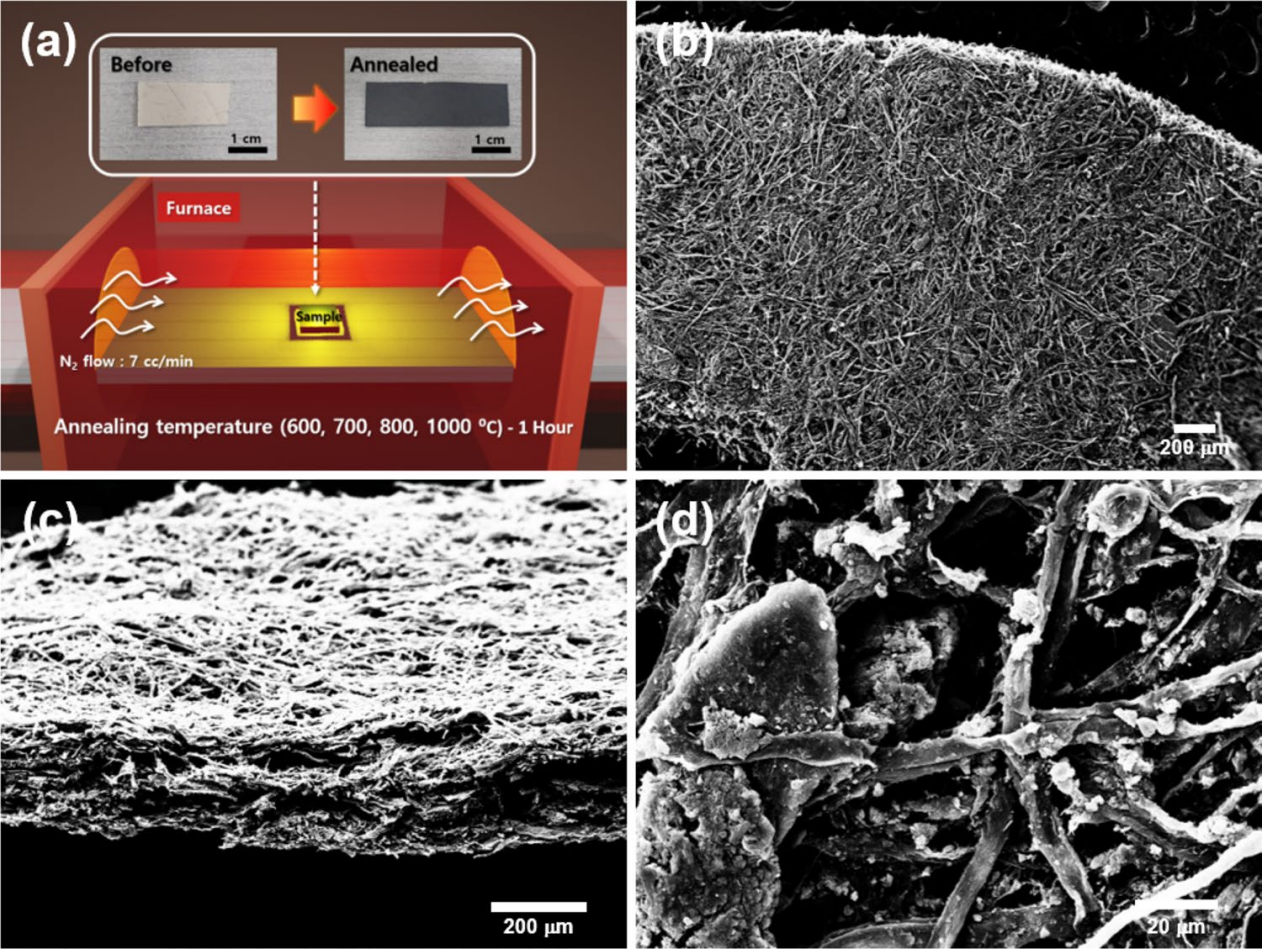
(**a**) Schematic image for annealing the paper substrate with various temperatures in N_2_ environment. Inset is photograph of the biochar before and after annealing [drawn by Blender (ver. 2.81) (https://www.blender.org/download/releases/2-81)]. (**b**–**d**) SEM images of the annealed biochar with 600 °C. (**b**,**c**) are top- and side-view of the biochar, respectively. (**d**) Fibril network and CaCO_3_ on the network are presented.

**Figure 2:**
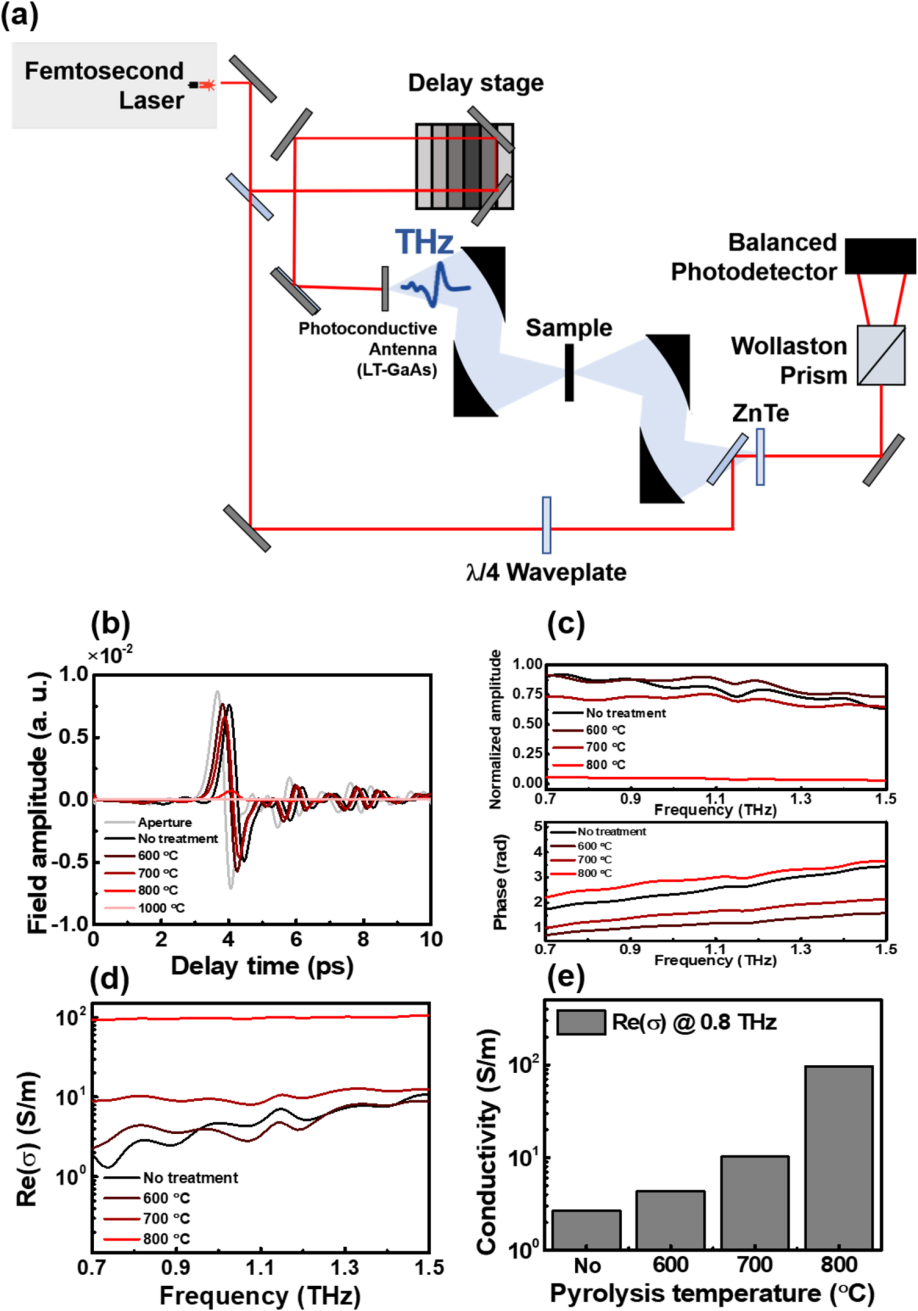
Terahertz time-domain spectroscopy of the paper substrate with and without annealing. (**a**) Experimental setup for terahertz time-domain spectroscopy. (**b**) Time trace of terahertz transmitted amplitude. (**c**) Normalized amplitude and phase of Fourier transformed data. (**d**) Frequency-dependent optical conductivity. (**e**) Summary of optical (at 0.8 THz) conductivity with annealing temperature.

The biochar sample was obtained by pyrolysis of a paper as shown in Fig. 1a. Detailed information on sample preparation is provided in “Methods” section. Figure 1b–d show scanning electron microscopy (SEM) images of annealed biochar sample at 600 °C, where the fibril network in the biochar is clearly shown. Note that the biochar can also be made as a powder on rigid substrates for practical applications such as terahertz shielding and metamaterials based on desired patterned conducting materials, as shown in Supplementary Information (Figs. S1, S2). We measured the terahertz transmission spectra and extracted frequency-dependent electrical conductivity of the biochar using a homemade terahertz time-domain spectroscopy system (Fig. 2a–e). The terahertz transmission and conductivity of the paper without annealing were also measured as a control experiment (black line). In Fig. 2b, one can check that the transmitted amplitude decreases as the annealing temperature increases. This phenomenon was also clearly shown in biochar powder samples, confirmed by terahertz imaging (Fig. S2). Especially, when the annealing temperature reaches 900 and 1000 °C, the transmitted amplitude is indistinguishable from the noise. Therefore, data only up to 800 °C were used for subsequent analysis. Normalized amplitude and phase of Fourier transformed data were shown in Fig. 2c. Still, the normalized amplitude decreases as the annealing temperature increases. We note that resonances due to the absorption of low-energy photons, such as intermolecular vibrations, were not observed. Since the terahertz transmission is sensitive to the electrical conductivity of a material, it can be inferred that the decrease in the transmission is due to an increase in the electrical conductivity of the material. This hypothesis can be confirmed directly using the equations obtained in “Methods” section. Obtained terahertz electrical conductivity was shown in Fig. 2d,e. Surprisingly, the terahertz conductivity of the biochar increased exponentially with increasing annealing temperature. When the annealing temperature was 600 °C, the electrical conductivity of the biochar was only a few S/m. However, as the annealing temperature increased up to 700 °C and 800 °C, the electrical conductivity increased significantly to the order of 10 S/m and 10^2^ S/m.

**Figure 3:**
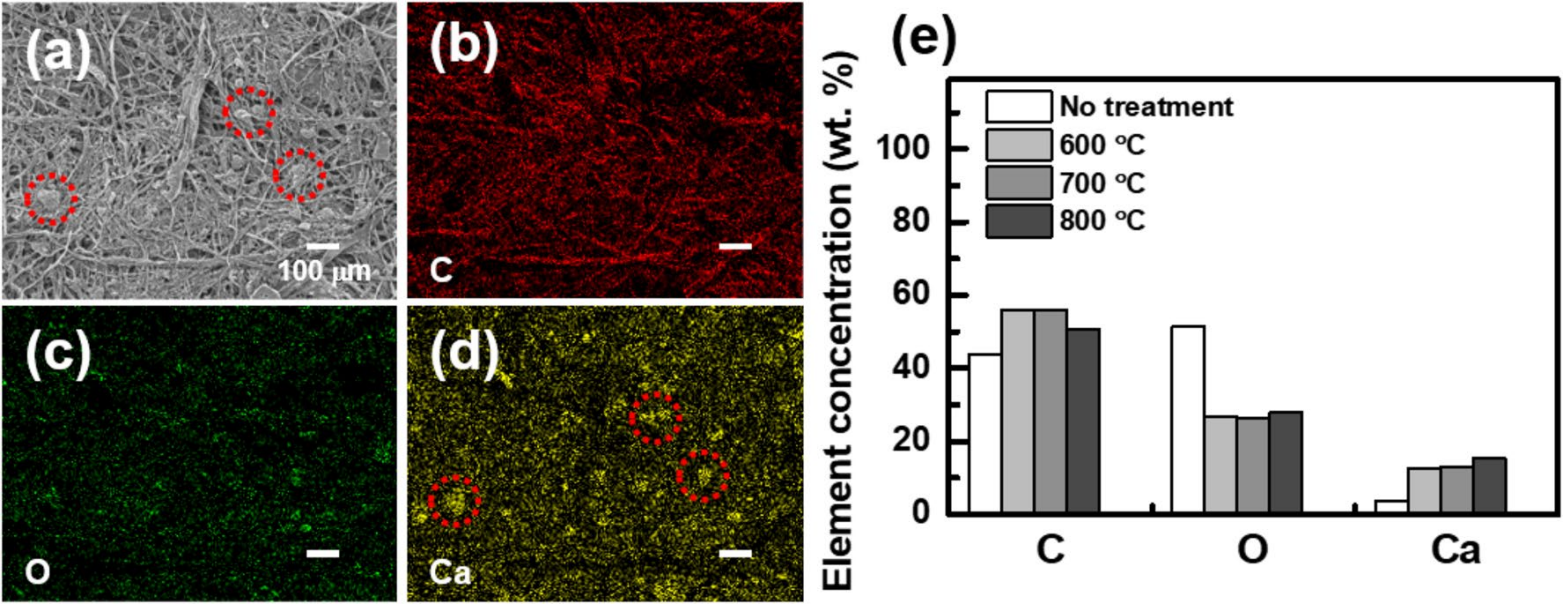
(**a**) SEM image and the corresponding EDS results for (**b**) carbon, (**c**) oxygen, and (**d**) calcium. The fibril structure matches with carbon distribution. The red-dotted circles in (**a**) and (**d**) show the overlap of the particles and calcium distribution. (**e**) Summary for the annealing-temperature-dependent element concentration.

To understand the origin of becoming a conducting material, now we move on to elemental and structural analysis for supporting the electrical results. First of all, we carried out an energy-dispersive X-ray spectroscopy (EDS) experiment as shown in Fig. 3. It was confirmed that the constituent elements of the biochar are mainly carbon, oxygen, and calcium. Figure 3b–d show the EDS mapping image for each element, corresponding to the SEM image shown in Fig. 3a. The distribution of carbon mainly overlapped with the fibril network of the biochar as in Fig. 3a. On the other hand, the distribution of calcium was mainly overlapped with the distribution of particles shown in Fig. 3a (red-dotted circle). Oxygen was distributed relatively evenly. The observation is consistent with the fact that paper is composed of various materials such as cellulose^13^ and CaCO_3_^14^ (which is commonly used as a fertilizer), and these are mainly composed of carbon, oxygen, and calcium. That is, the difference in the distribution of each element can be understood as a result of the different distribution of substances (cellulose, CaCO_3_, etc.).

The relative weight concentrations of each element in the biochar are summarized in Fig. 3e and Table 1. After the annealing process, the overall concentration of carbon and calcium increased, while the concentration of oxygen decreased. This can be understood through the process of carbonization. During the pyrolysis process, the moisture in the paper substrate volatilizes^6^. Also, cellulose begins to degrade at 315–400 °C^15^. At this temperature, the oxygen concentration becomes lower and the concentrations of carbon and calcium become relatively higher. It is noteworthy that the carbon (calcium) concentration gradually decreases (increases) as the annealing temperature increases. As the degradation of cellulose continues, all the volatile materials (such as CO_2_, CH_4_, CO, etc.) also begin to be removed^6,15^. Some carbon disappears in this situation, but calcium still remains. This process becomes faster with higher annealing temperatures. Consequently, the concentration of carbon slightly decreases, and the calcium concentration increases in the annealing process with higher temperatures.

**Table 1:** EDS data of biochar samples with different pyrolysis temperature.

Pyrolysis temperature	C		O		Mg		Al	
	wt%	at%	wt%	at%	wt%	at%	wt%	at%
No treatment	43.8	52.11	51.55	46.04			0.80	0.42
600 °C	56.06	68.47	26.54	24.33	0.25	0.15	2.75	1.49
700 °C	56.03	68.58	26.2	24.08	0.27	0.16	2.85	1.55
800 °C	50.74	64.30	27.94	26.58	0.34	0.21	3.25	1.84
1000 °C	56.02	69.18	24.49	22.71	0.37	0.23	2.8	1.54

Pyrolysis temperature	Si		S		Ca			
	wt%	at%	wt%	at%	wt%	at%		
No treatment	0.39	0.20			3.46	1.23		
600 °C	1.61	0.84	0.4	0.18	12.40	4.54		
700 °C	1.63	0.85			13.02	4.77		
800 °C	1.83	0.99	0.44	0.21	15.44	5.86		
1000 °C	1.71	0.90	0.35	0.16	14.26	5.28		

**Figure 4:**
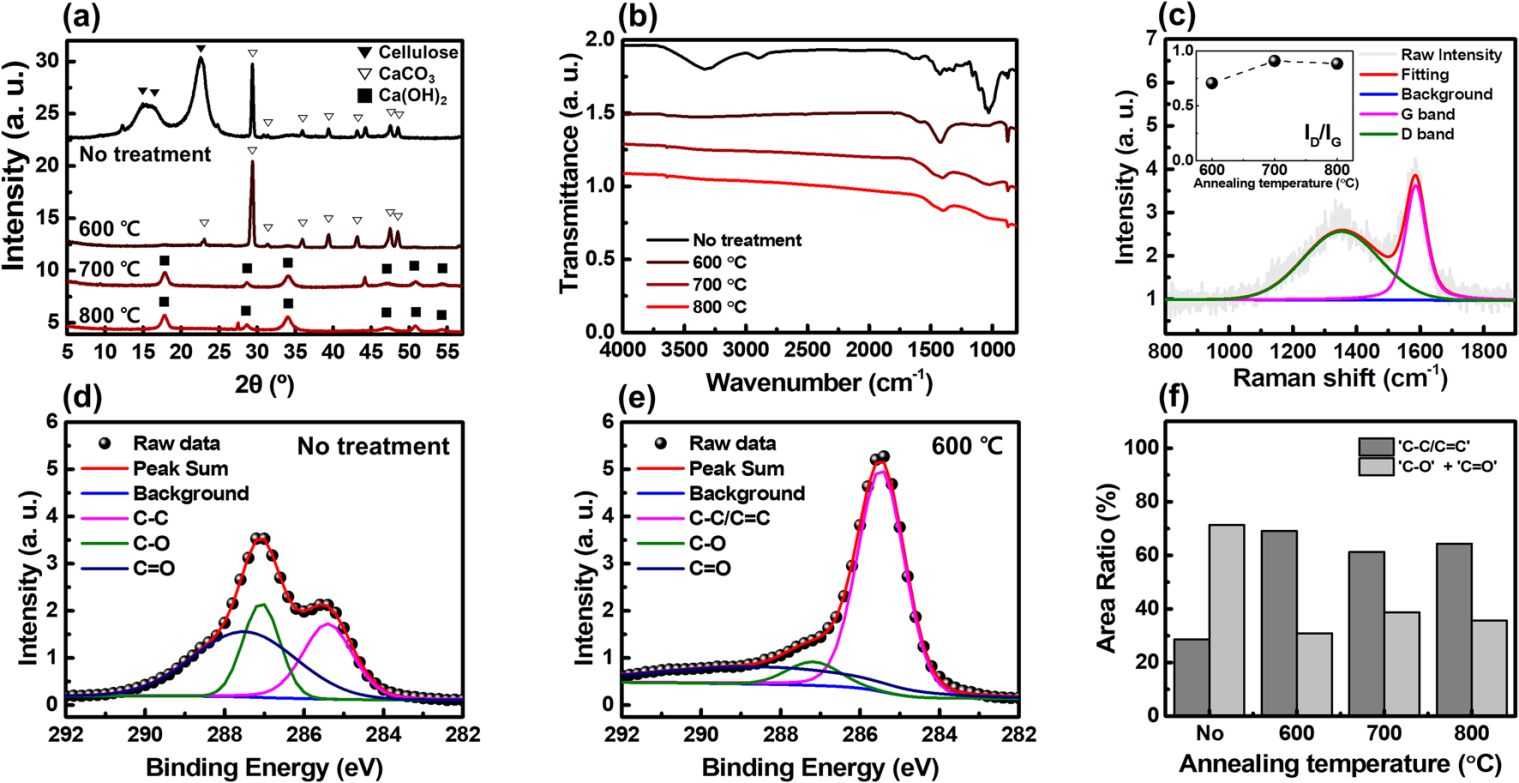
Structural analysis such as (**a**) XRD, (**b**) FT-IR, (**c**) Raman spectroscopy, and (**d**–**f**) XPS results, respectively.

Based on the data in Table 1, we measured and analyzed the X-ray diffraction (XRD), Fourier-transform infrared spectroscopy (FT-IR), Raman, and X-ray photoelectron spectroscopy (XPS) spectra as shown in Fig. 4. Figure 4a shows the XRD data of each sample. In the case of the paper substrate, only peaks related to cellulose^16^ and CaCO_3_^17^ were observed. On the other hand, in the case of the 600 °C-annealed-sample, no cellulose-related picks were observed, while CaCO_3_-related picks were still observed. When the annealing temperature increased up to 700 °C or 800 °C, the calcium-carbonate-related peaks disappeared, and instead, calcium-hydroxide-related peaks were observed^18^. Also, no carbon-related peaks were observed. The thermal decomposition of CaCO_3_ is known to be initiated around 700–800 °C^19^. At this temperature, CaCO_3_ releases carbon dioxide and becomes calcium oxide^20^. After that, calcium oxide is combined with water vapor in the air to become calcium hydroxide^19^. During this process, some carbon is volatilized, reducing the relative weight concentration in the entire sample. These XRD results are consistent with the EDS results summarized in Fig. 3e. We may attribute the intensity of the carbon-related peak, which is relatively weak compared to that of the calcium-related materials, to the low crystallinity of the pyrolyzed paper.

Figure 4b shows the FT-IR spectra of each sample. It can be seen that the spectra of the biochar samples and the paper substrate are significantly different. Two broad absorption bands are observed in the spectrum of the paper substrate: 3730–2800 cm^–1^ and 1700–800 cm^–1^. On the other hand, all these peaks disappear from the spectra of the biochar samples. In the biochar samples, peaks are mainly observed at 1400 cm^–1^ and 875 cm^–1^, and if the annealing temperature exceeds 600 °C, a small peak is also observed near 3642 cm^–1^. In the spectrum of the paper substrate, the absorption peak at 3340 cm^–1^ is mainly due to the O–H stretch bond, while the peak at 2898 cm^–1^ is attributed to the C–H stretch bond in cellulose^21^. In addition, the peak at 1162 cm^–1^, 1106 cm^–1^, 1049 cm^–1^, and 1029 cm^–1^ are attributed to the asymmetric C–O–C bridge stretch bond^22^, anhydroglucose ring^22^, C–O–C pyranose ring skeletal vibration^23^, and C–O stretch bond^24^, respectively. These peaks indicate that the paper substrate is mainly composed of cellulose. In the spectrum of the biochar samples, the broad absorption peak around 1400 cm^–1^ and a sharp peak at 874 cm^–1^ are attributed to the vibrations of ${\mathrm{CO}}_{3}^{-2}$^25^. The small and sharp pick at 3642 cm^–1^ is due to the O–H stretching vibration in calcium hydroxide^26^. Legodi et al*.* demonstrated that the peak at 874 cm^–1^ in the FT-IR spectra could be a quantitative tool for measuring the ratio of CaCO_3_/Ca(OH)_2_ mixtures^26^. According to previous literature^26^, the peak intensity increases as the proportion of CaCO_3_ in the mixture increases. In our experiments, the peak intensity gradually decreases as the annealing temperature increases. That is, as the annealing temperature increases, the rate at which CaCO_3_ is decomposed into calcium hydroxide increases. This argument can also be supported by an increase of the peak intensity at 3642 cm^–1^, which is attributed to the O–H stretching vibration of the calcium hydroxide. This result is consistent with the XRD spectra shown in Fig. 4a. It should be noted that the baseline of the FT-IR spectra gradually slopes down as the annealing temperature increases. This is a unique property observed when carbon materials such as biochar and carbon black are measured by attenuated total reflection-Fourier transform infrared spectroscopy (ATR-FTIR)^27^. When using ATR-FTIR, light absorption from carbon materials becomes greater with deeper light penetration at the lower wavenumber. This trend becomes greater as the absorption coefficient (or real part of the complex conductivity $Re(\stackrel{\sim }{\sigma }(\omega ))$) increases. That is, it shows that the optical conductivity of the biochar increases as the annealing temperature increases.

Figure 4c exhibits the Raman spectrum of 600 °C-annealed sample. In the case of the paper substrate, no significant spectrum was obtained. While, typical Raman spectra of carbon-based materials were observed in the biochar samples: e.g. D and G band near 1353 cm^–1^ and 1587 cm^–1^, respectively. The intensity ratio between D and G band in the inset of Fig. 4c can be employed to estimate the crystallinity degree of the carbon-based materials. The observed I_D_/I_G_ < 1 for all annealed cases indicates that the annealing process promotes crystallization of the paper substrate^28^.

Finally, we performed XPS experiments as shown in Fig. 4d–f. Figure 4d,e show the XPS data of the paper substrate and 600 °C-annealed sample, respectively. As mentioned above, paper substrates are mainly composed of cellulose. Therefore, it can be seen that the XPS spectrum of the paper substrate is consistent with the XPS spectrum of the cellulose^29–32^. When the paper substrate is pyrolyzed to become biochar, the ratio of C–O bonds and C=O bonds decreases, and the ratio of C–C bonds or C=C bonds increases by the carbonization process. The ratio of each bond depending on the annealing temperature is summarized in Fig. 4f. It is clearly shown that the ratio of C–C or C=C bond increased significantly in all biochar samples. That is, it can be seen that the carbon element remaining after the cellulose is pyrolyzed undergoes a crystallization process. This result is consistent with the Raman spectra shown in Fig. 4c. To summarize, by examining the various spectroscopic properties of the biochar, we have shown that the terahertz conductivity enhancement of the biochar was mainly originated from the pyrolysis of cellulose and the crystallization of carbon elements.

## Conclusion

In conclusion, we have demonstrated that the terahertz electrical conductivity of biochar obtained from paper substrate increases dramatically with increasing the annealing temperature. It was confirmed that the origin of the conductivity enhancement is mainly due to the crystallization of carbon elements after the cellulose in the paper substrate is thermally decomposed. As the annealing temperature increases, pyrolysis and crystallization proceed faster, resulting in higher electrical conductivity of the biochar. Our research shows that biochar, an eco-friendly, and sustainable material has a great potential to be used in paper-based devices operating at terahertz frequencies.

## Methods

### Materials

We used Elephant Poopoopaper (PoopoopaperTM) which is made in a chemical-free way and is environmentally friendly. The paper was placed in a furnace under a N_2_ environment and the furnace was heated up to 600, 700, 800, 900, and 1000 °C at a rate of 5 °C/min, respectively. Each sample was annealed for 1 hour. After the annealing process, the color changed from white to black as shown in the inset of Fig. 1a. No particular difference in surface flatness was found, as shown in Supplementary Information (Fig. S3). Note that calcium carbonate (CaCO_3_) particles are also shown on the network, which was discussed in “Results and discussion” section.

### Terahertz time-domain spectroscopy

We used Ti:sapphire femtosecond laser and low-temperature-grown GaAs photoconductive antenna to generate terahertz pulses. The center wavelength, the repetition rate, and the pulse width of the laser are 800 nm, 80 MHz, and 100 fs, respectively. The terahertz pulses are focused with parabolic mirrors and normally incident on the biochar. Transmitted terahertz waves are collected by parabolic mirrors and detected via electro-optic sampling method using a (110)-oriented 1-mm-thick ZnTe crystal. The time-domain waveforms are converted to frequency spectra using a numerical Fourier transform. The terahertz spectra of the biochar are normalized to the spectra from the air.

### Calculations of the complex refractive index and the complex conductivity

The relation between the normalized spectra and the complex refractive index of the biochar can be expressed as follows^33^:

$$\tilde{t }(\omega )=\rho \left(\omega \right) \cdot exp[i\theta \left(\omega \right)]=\frac{4\tilde{n }(\omega )\cdot exp\left[i\left(\tilde{n }(\omega )-1\right){k}_{0}h\right]}{{\left(\tilde{n }(\omega )+1\right)}^{2}}$$

where *ρ*(*ω*) is an amplitude, *θ*(*ω*) is a phase of the normalized spectra, $\tilde{n }\left(\omega \right)=n\left(\omega \right)+i\kappa (\omega )$ is a complex refractive index of the biochar, *ω* is the angular frequency, *c* is light velocity, and *h* is a thickness of the biochar, respectively. From this equation, we extracted the complex refractive index $\tilde{n }(\omega )$ by using the numerical method. Then, from the relation^34^

$$\tilde{n }(\omega )=\sqrt{\stackrel{\sim }{\varepsilon }(\omega )}=\sqrt{1+\frac{i\stackrel{\sim }{\sigma }(\omega )}{{\varepsilon }_{0}\omega }}$$

where $\stackrel{\sim }{\varepsilon }(\omega )$ is a complex dielectric function, ${\varepsilon }_{0}$ is a vacuum permittivity, $\stackrel{\sim }{\sigma }(\omega )$ is a complex conductivity, $\stackrel{\sim }{\sigma }(\omega )$ can be expressed as $\stackrel{\sim }{\sigma }(\omega )=-i{\upvarepsilon }_{0}\omega \left({\tilde{n }}^{2}(\omega )-1\right)$. Therefore, the real part of the terahertz conductivity $Re(\stackrel{\sim }{\sigma }(\omega ))$ is determined by $Re\left(\stackrel{\sim }{\sigma }(\omega )\right)=2n(\omega )\kappa (\omega ){\varepsilon }_{0}\omega$.

### Structural characterization

The morphology of the biochar samples and their energy-dispersive X-ray spectra (EDS) were obtained using field emission scanning electron microscopy (JSM-7800F, JEOL). The crystallinity and compositions of the biochar were characterized by X-ray diffraction (XRD) using a high-resolution X-ray diffractometer (SmartLab, Rigaku) with Cu Kα radiation. Elemental compositions of the biochar were also analyzed using X-ray photoelectron spectroscopy (XPS) (PHI 5000 VersaProbe II, ULVAC-PHI). In addition, Fourier-transform infrared spectroscopy (FT-IR) (VERTEX 80v and Hyperion 2000, Bruker) and Raman spectroscopy (Xper Ram 200, Nanobase) using a 532 nm laser were also performed to analyze the molecular structure of the biochar.

## Supplementary Information

Supplementary Information 1.
