# Supplementary material for: Biochar as a low-cost, eco-friendly, and electrically conductive material for terahertz applications
Source: Sci Rep. 2021 Sep 16;11:18498. doi: 10.1038/s41598-021-98009-5 (PMC8445971; doi:10.1038/s41598-021-98009-5)
Supplement: Supplementary file 1 — Supplementary Information 1. [file 41598_2021_98009_MOESM1_ESM.docx]

**Supporting Information**

Biochar as a low-cost, eco-friendly, and electrically conductive material for terahertz applications

# Woongkyu Park^1†^, Hyuntae Kim^2†^, Hajung Park^2^, Soobong Choi^1,2*^, Sung Ju Hong^3*^, and Young-Mi Bahk^1,2*^

^1^Intelligent Sensor Convergence Research Center (ISCRC), Incheon National University, Incheon 22012, Republic of Korea

^2^Department of Physics, Incheon National University, Incheon 22012, Republic of Korea

^3^Division of Science Education, Kangwon National University, Chuncheon 24341, Republic of Korea

^*^E-mail: sbchoi@inu.ac.kr, sjh@kangwon.ac.kr, ymb@inu.ac.kr

1. **Biochar powder on rigid substrates.**

To explore the potential of the biochar for practical applications, paper and biochar powder were fabricated on rigid substrates (Figure S1). The paper powder was placed in a furnace and annealed with the conditions mentioned in the Method section.


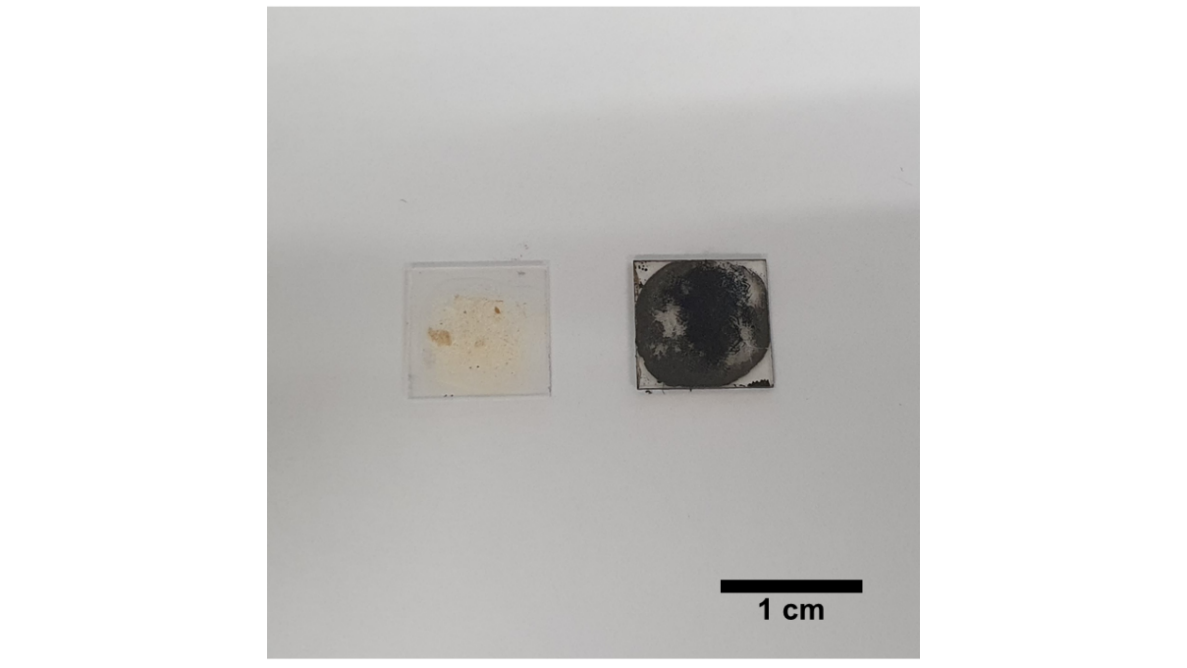


Figure S1. Photograph of paper and biochar powder on rigid substrates.

1. **Terahertz imaging of the biochar powder sample.**

As mentioned in the main article, the biochar can also be made as a powder form for practical applications such as terahertz shielding. To verify the possibility of such practical applications, terahertz imaging of the biochar powder sample was performed. In Figure S2 (a), photograph of biochar powder sample for terahertz imaging experiment is shown. The biochar powder was fabricated on sapphire substrate, and the annealing temperature was 500 ℃. As shown in Figure S2 (b), terahertz imaging experiment was performed in the area marked in Figure S2 (a). The decrease of transmitted amplitude at the biochar sample (yellow and green asterisks in the Figure S2 (a, b)) is clearly shown in constrast to the one at the bare substrate (blue asterisk in the Figure S2 (a, b)).


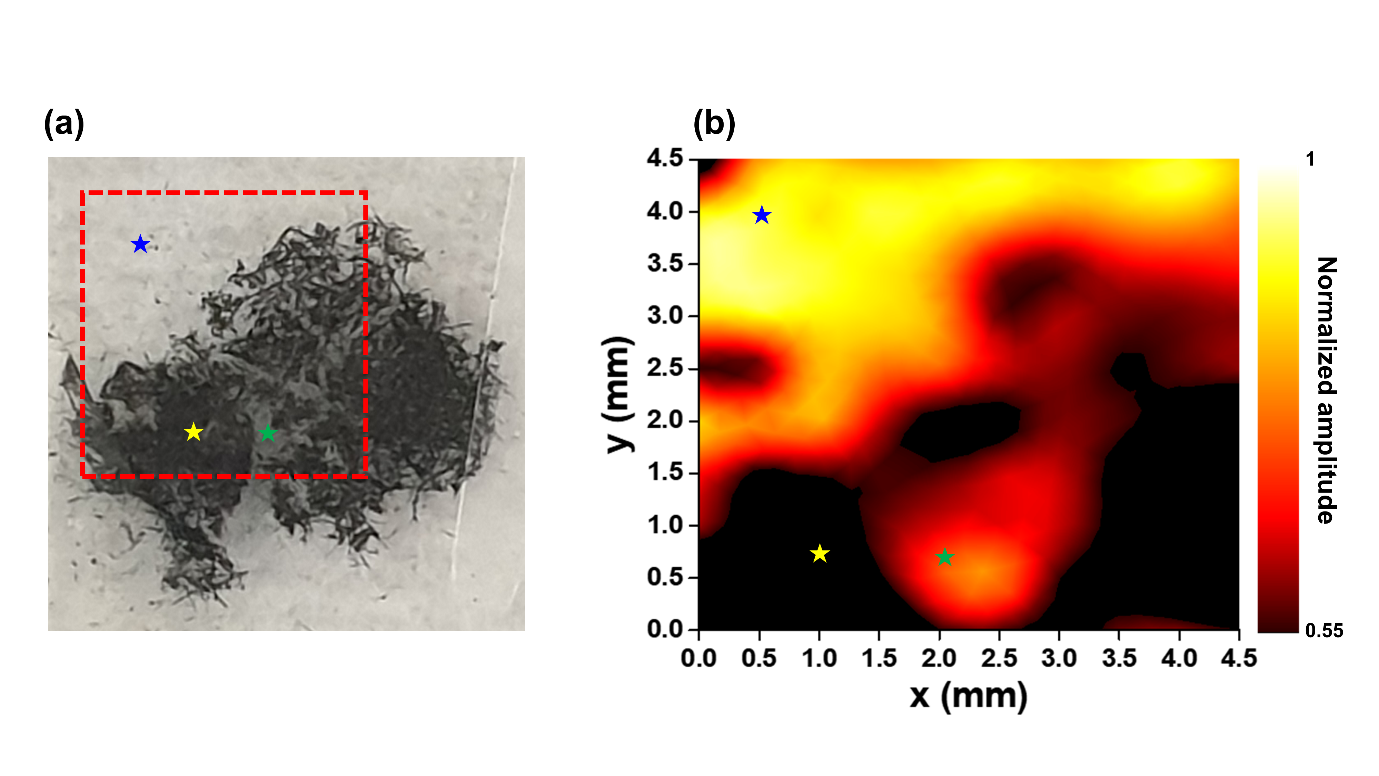


Figure S2. (a) Photograph of biochar powder sample for terahertz imaging experiment. The dashed line indicates imaged area. (b) Terahertz imaging data of the biochar powder. The blue asterisk indicates the substrate region, while the yellow and green asterisks indicate the biochar region.

1. **Surface flatness of the biochars.**

From a macroscopic point of view, the surface flatness of biochars before and after annealing is not significantly different, as shown in Figure 1(a). In Figure S3, SEM images of biochars before and after annealing are compared. In our observation, no particular difference in surface flatness was found.


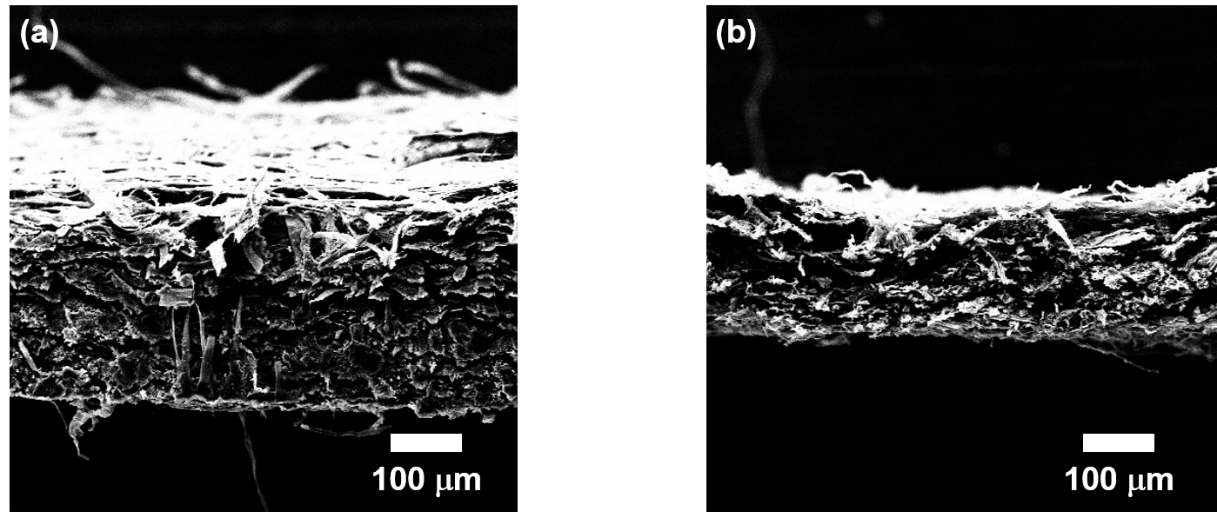


Figure S3. Cross-sectional SEM images of (a) the paper and (b) the annealed biochar.
